# Chloride of Iron and Chloride of Sodium as a Hæmostatic

**Published:** 1863-11

**Authors:** 


CHLORIDE OF IRON AND CHLORIDE OF SODIUM AS A HÆMOSTATIC.—Dr. Piazza of Bologna, has introduced a new preparation containing chloride of iron, which he has found very efficacious as an hæmostatic agent. You are aware that a solution of chloride of iron has already been used successfully for stopping the flow of blood from wounds, but in a concentrated state it often causes much pain and irritation. It appears that when chloride of sodium is added to it, the proportion of chloride of iron may be considerably diminished. The solution Dr. Piazza recommends is composed of perchloride of iron having a specific gravity of 1.075 to 1.11, mixed with an equal volume of a concentrated solution of chloride of sodium. Great success seems to have attended the use of this solution in Italy and Belgium. The chloride of iron should have no free acid in it.—*Medical and Surg. Rep.*

